# Human metallo-β-lactamase enzymes degrade penicillin

**DOI:** 10.1038/s41598-019-48723-y

**Published:** 2019-08-21

**Authors:** Seydina M. Diene, Lucile Pinault, Vivek Keshri, Nicholas Armstrong, Saber Khelaifia, Eric Chabrière, Gustavo Caetano-Anolles, Philippe Colson, Bernard La Scola, Jean-Marc Rolain, Pierre Pontarotti, Didier Raoult

**Affiliations:** 1 Aix Marseille Université, MEPHI, IHU-Méditerranée Infection, Marseille, France; 2 Assistance Publique-Hôpitaux de Marseille (AP-HM), IHU-Méditerranée Infection, Marseille, France; 3 IHU-Méditerranée Infection, Marseille, France; 4 Evolutionary Bioinformatics Laboratory, Department of Crop Sciences, University of Illinois at Urbana-Champaign, Urbana, IL 61801 USA; 5 CNRS, Marseille, France

**Keywords:** Clinical microbiology, Genetics research

## Abstract

Nonribosomal peptides are assemblages, including antibiotics, of canonical amino acids and other molecules. β-lactam antibiotics act on bacterial cell walls and can be cleaved by β-lactamases. β-lactamase activity in humans has been neglected, even though eighteen enzymes have already been annotated such in human genome. Their hydrolysis activities on antibiotics have not been previously investigated. Here, we report that human cells were able to digest penicillin and this activity was inhibited by β-lactamase inhibitor, i.e. sulbactam. Penicillin degradation in human cells was microbiologically demonstrated on *Pneumococcus*. We expressed a MBLAC2 human β-lactamase, known as an exosome biogenesis enzyme. It cleaved penicillin and was inhibited by sulbactam. Finally, β-lactamases are widely distributed, archaic, and have wide spectrum, including digesting anticancer and β-lactams, that can be then used as nutriments. The evidence of the other MBLAC2 role as a *bona fide* β-lactamase allows for reassessment of β-lactams and β-lactamases role in humans.

## Introduction

Nonribosomal peptides and polyketide synthases are assemblages of amino acids and other molecules that may reflect the pre-ribosomal synthesis of peptides. First identified in fungi and bacteria and then used as antibiotics^1^ or anticancer drugs, it is now possible to identify them in most living organisms; even springtail hexapods secrete penicillin^2^. As part of our works on antibiotic resistance, we have previously demonstrated that ancient β-lactamase reconstituted from metagenomic datasets can be effective on current antibiotics such as penicillin^3^. Currently, more than 1,600 bacterial β-lactamases have been described and classified into four molecular classes, labelled A, B, C and D^4^. While three classes, A, C, and D, use a catalytically active serine residue for the inactivation of the β-lactam drug, class B (metallo-enzymes), which requires zinc as a cofactor for their catalytic activity, is the most heterogeneous class and exhibits highly conserved motifs in its catalytic sites. Class B enzymes belong to the large superfamily of metallo-β-lactamase fold proteins. This superfamily includes more than 34,000 proteins with diverse functions, including β-lactamases, flavoproteins, glyoxalase II, arylsulfatases, cyclases, alkyl sulfatases, CMP-NeuAc hydroxylases, cAMP phosphodiesterases, DNA cross-link repair proteins, cleavage and polyadenylation specific factors, phosphonate metabolism proteins, ribonucleases and choline binding proteins^5–8^. Class B enzymes, presenting an archaic fold, are widely distributed in nature and have been described in all domains of life, including bacteria, archaea and eukaryotes, including human^6^. Interestingly, described human metallo-β-lactamases exhibited catalytic sites able to bind metal ions including zinc and iron (as seen in classical bacterial metallo-β-lactamases) to catalyze a wide range of chemically reactions^8^. Thus, according to that, β-lactamase activity of genes, annotated as such in human cells, is becoming crucial for us to understand their role.

## Results

### Human metallo-β-lactamases (hMBLs)

Metallo-β-lactamases in eukaryotic cells, including human cells, were first annotated a long time ago^9^. They appeared as part of polycistronic genes, whose activities in humans have thus far mostly focused on their anti-ribonuclease activities, which contribute to the rapid degradation of unnecessary RNA messengers or DNA/RNA interacting mechanisms, or biochemical mechanisms, especially in mitochondria^8–10^. However, despite the fact that eighteen human metallo-β-lactamase enzymes have been reported in the literature^8^ and that some of them (e.g., SNM1 enzymes) are active on chemotherapeutic agents, such as cisplatin or mitomycin^8^, their activities on β-lactam antibiotics have, to the best of our knowledge, never been investigated. Indeed, our analyses revealed that six of these 18 human metallo-ß-lactamases (i.e. LactB2, MBLAC1, MBLAC2, SNM1A, SNM1B, and PNKD1) exhibited the conserved bacterial metallo-ß-lactamases motif “HxHxDH” and histidine residues (H196 and H263) (Supplementary Fig. 1). four of these six human metallo-ß-lactamases including, LactB2, acting as an endoribonuclease^11^, MBLAC2, involved in B-cell exosome biogenesis^12^, SNM1A and SNM1B, acting as DNA cross-link repair proteins, were analysed by three-dimensional (3D) comparison against the Phyre2 investigator database. This comparison revealed 100% confidence and more than 92% coverage with the crystal structure of the β-lactamase domain protein from *Burkholderia ambifaria* (Phyre2 ID: C5i0pB) for the MBLAC2 enzyme, while LactB2, SNM1A and SNM1B have already been characterized in this database as human β-lactamase-like protein, human dna cross-link repair 1a, and human 5′ exonuclease respectively (Supplementary Table 1).

### Monitoring β-lactamase activities in human cells

**Figure 1:**
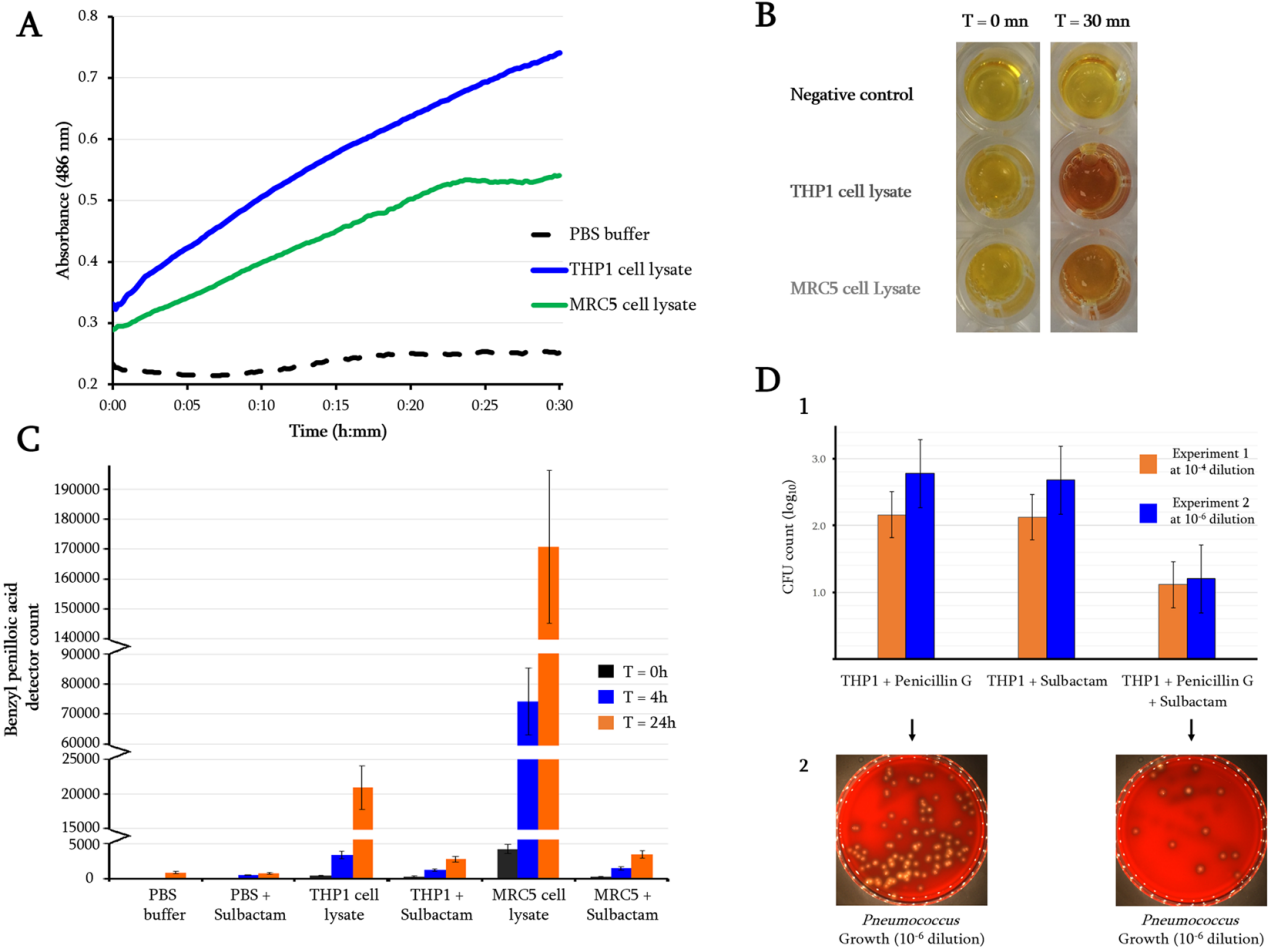
Beta-lactamase activity detected in human cell cultures. (**A**) Spectrophotometric assay of nitrocefin degradation in the presence of THP1 and MRC5 cell lysates monitored for 30 minutes by monitoring absorbance at 486 nm; (**B**) Nitrocefin solutions with added THP1 and MRC5 cell lysates or PBS (used as negative control), incubated at 25 °C and observed after 30 minutes. The appearance of a red-coloured product indicates positive β-lactamase activity, while a constant yellow colour indicates no activity; (**C**) LC/MS average detector counts of metabolite benzyl penilloic acid monitored during 24 hours. Penicillin G was added in the presence of human cell lysates (THP1 and MRC5) in the presence or absence of sulbactam; (**D**) determination of the growth of highly penicillin-susceptible *Pneumococcus* within human THP1 cells in the presence of penicillin G or sulbactam alone, and in the presence of both. (**D1**) CFU count (log_10_) of the *Pneumococcus* strain under the three different conditions after 24 h incubation in THP1 cells. (**D2**) Bacterial colonies resulting from THP1 cell lysis grown on agar plates and observed after 24 hours. The *Pneumococcus* strain grew more easily within THP1 cells, despite the presence of penicillin G, than when sulbactam was added.

The functionality of human metallo-β-lactamases on β-lactam drugs appears to be a highly significant question since penicillin has been described as being inactive against obligate intracellular bacteria, such as *Coxiella spp*., *Rickettsia spp*., *Anaplasma spp*. and *Ehrlichia spp*.^13–15^. This activity has been considered as the consequence of either a limited uptake of the drug because of passive penetration through cell membranes or antibiotic degradation by cellular enzymes^13,16^. However, some β-lactams, such as third generation cephalosporins, which resist most β-lactamases, are active on intracellular *C*. *burnetii*^17^. We have evaluated the degradation of penicillin G by THP1 and MRC5 human cells. At the concentration of 10^6^ cell/ml, THP1 human cell lysates were able to significantly hydrolyse penicillin G (Supplementary Fig. 2E,F) and nitrocefin (Fig. 1A,B). Interestingly, monitoring of penicillin G degradation toward its metabolite, i.e., benzyl penilloic acid, proves that THP1 and MRC5 human cells are able to hydrolyse penicillin G following the well-described alkyl pH degradation pathway^18^, and this hydrolysis activity was inhibited in the presence of β-lactamase inhibitors, i.e. sulbactam in both human cell types (Fig. 1C). The same inhibition effect in human cells was also observed using another β-lactamase inhibitor i.e. clavulanic acid (data not shown). Moreover, we were able to show that a highly penicillin-susceptible *Pneumococcus* strain could grow more easily within human THP1 cells in the presence of penicillin G alone than when the β-lactamase inhibitor (sulbactam) was added (Fig. 1D).

**Figure 2:**
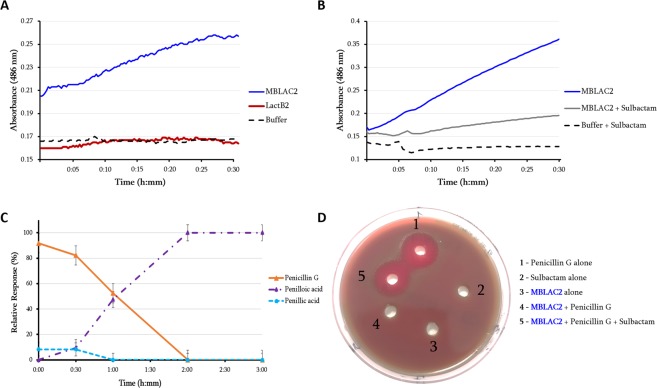
Characterization of two hMBL enzymes. (**A**) Spectrophotometric assay of nitrocefin degradation in the presence of MBLAC2 and LactB2 enzymes, showing the real hydrolysis activity of MBLAC2, whereas LactB2 exhibits no activity on this substrate; (**B**) monitoring nitrocefin degradation over time in the presence and absence of sulbactam (1 µg/ml). (**C**) LC/MS average relative response of the screened metabolite compounds of penicillin G in the presence of the MBLAC2 hMBL enzyme monitored for three hours. Penicillin G (in orange) refers to the intact form of the antibiotic and penilloic acid (in purple) and penillic acid (in light blue) refer to the penicillin G metabolites. Penicillin G in PBS did not show any degradation to any metabolite (data not shown). (**D**) Microbiological test of the mixture of penicillin G (0.1 µg/ml) with the human β-lactamase enzyme (MBLAC2) in the presence and absence of sulbactam on the highly penicillin-susceptible and sulbactam-resistant *Pneumococcus* strain. The halo around holes 1 and 5 reveals the growth inhibition of the *Pneumococcus* strain. The absence of this halo around holes 2, 3, and 4 indicates that no effect of the mixture on *Pneumococcus* growth was observed.

Among the six human metallo-ß-lactamases with signatures similar to those of bacterial β-lactamases, four human metallo-ß-lactamases (LactB2, MBLAC2, SNM1A, and SNM1B) were tested for β-lactamase activity. They were cloned and expressed in *Escherichia coli*, and the purified proteins were evaluated. While no β-lactamase activity was detected for the LactB2 enzyme (Fig. 2A), β-lactam hydrolysis on nitrocefin was detected for the MBLAC2 enzyme (Fig. 2A,B) as well as for penicillin G (Fig. 2C). Inhibition of this β-lactamase was observed in the presence of sulbactam (Fig. 2B). Furthermore, this effect was confirmed microbiologically by the growth of a highly susceptible *Pneumococcus* strain in the presence of 0.1 µg/ml penicillin G incubated with MBLAC2, which was inhibited by sulbactam (Fig. 2D). On the basis of the enzymatic characterization that was performed with data fitted to the Michaelis-Menten equation (R^2^ = 0.967) giving kinetic parameters k_cat_ = 4.15 × 10^−4^ s^−1^, K_M_ = 370.5 µM and resulting k_cat_/K_M_ = 1.12 s^−1^.M^−1^ (Supplementary Table 2), optimizing the conditions for efficacy of these β-lactamases could improve their hydrolysis activity *in vitro*. Moreover, to confirm the specific β-lactam hydrolysis activity by these human metallo-β-lactamases, soluble domains containing the MBL motif of SNM1A and SNM1B were expressed and tested on both nitrocefin and penicillin G. As shown on Supplementary Fig. 3, nitrocefin substrate was degraded by both enzymes even if the SNM1B enzyme appeared more active. The hydrolysis test on penicillin G by LC-MS shows, as expected, a significant hydrolysis activity for both enzymes and these latter were also inhibited by the β-lactamase inhibitor i.e. sulbactam (Supplementary Figs 4 and 5).

Unfortunately, the activity of these three human enzymes (MBLAC2, SNM1A, and SNM1B) were tested on the other β-lactam antibiotic classes including cephalosporins (cefotaxime) and carbapenems (imipenem) but no activity was detected against these antibiotics. These results were confirmed by the susceptibility testing assay of the transformed *E*. *coli* BL21 strain which remains susceptible to these antibiotics.

## Discussion

The source and ancestrality of β-lactamase enzymes are important points to consider. Indeed, β-lactams, like most antibiotics, are part of the microorganisms’ arsenal in their struggle to control microbial ecosystems^19^. Most of these antibiotics are metabolic products of non-ribosomal peptide synthetases and polyketide synthetases, the only translation systems outside the ribosome that have structural motifs^20,21^. Most living organisms have nonribosomal peptides, polyketide synthases, or hybrids^22,23^, which may be destroyed by metallo-β-lactamases. Investigation of the putative ancestrality of β-lactamase motifs, as seen by Caetano *et al*., shows that they possess among the oldest enzymatic motifs on the earth^21^. The existence of natural β-lactamases in eukaryotes, bacteria, and archaea highlights the fact that β-lactam may have multiple roles, as well as broad activity, that may target nonribosomal peptides from different organisms, such as mitochondria. Moreover, β-lactams can be used by bacteria as nutriment after β-lactamase cleavage^24,25^. These roles should be investigated, especially in humans, as they have already been found to be involved in resistance to anticancer drugs^8^. Activity as β-lactamase and its inhibition by β-lactamase inhibitors may be great significant for humans, since these expressed enzymes by genes identified as β-lactamase fold have many activities which some of them (e.g. SNM1A, MBLAC1, or Artemis), are extremely important for life of vertebrates. Indeed, it has been shown that these metallo-β-lactamase fold enzymes, involved in DNA repair mechanism, are inhibited by ceftriaxone and moreover β-lactamase inhibitors and β-lactams resistant to β-lactamases can alter expression of proteins, especially human proteins^26,27^, whose role have nothing to do with penicillin degradation. β-lactamase-resistant cephalosporins result in a downward regulation of repair nucleases that have a metallo-β-lactamases fold^26^.

In conclusion, we demonstrate here, for the first time, that human cells are able to hydrolyse penicillin antibiotics through metallo-β-lactamase fold enzymes. We show that three tested of these enzymes, i.e. MBLAC2, SNM1A, and SNM1B actively hydrolyse penicillin β-lactam class, and that this activity is inhibited by β-lactamase inhibitors in both *in vitro* experiments and a human cell model. These findings open new therapeutic avenues and bring new insights into the use of antibiotics for the treatment of infections caused by obligate intracellular bacteria.

## Materials and Methods

### Sequence analysis

The uncharacterized MBLAC2 (NP_981951) enzyme, reported as being involved in B-cell exosome biogenesis, the LactB2 (Q53H82) enzyme described as an endoribonuclease, as well as exonucleases SNM1A (Q6PJP8) and SNM1B (Q9H816) were selected to investigate their putative hydrolysis activity on β-lactam antibiotics. The nucleotide sequences of these selected genes, were synthesized and cloned into the pET24a(+) expression plasmid. Moreover, β-lactam hydrolysis activity was investigated directly in two human cells, THP1, a human monocytic cell line and MRC5, a human fibroblast cell line.

### β-lactamase activity evaluation in human cells

To evaluate the β-lactamase activity in human cells, 10^6^ cell/ml of THP1 cells were incubated on R10 + 2 medium with 2 µg/ml of penicillin for three days. Both 10 μl of the supernatant and the pellet from the THP1 cell culture were then deposited in holes created on agar medium. A clinical highly penicillin-susceptible *Streptococcus pneumoniae* strain (MIC_PeniG_ = 0.012 µg/ml) was plated on agar medium and incubated for 24 hours. β-lactamase activity, i.e. ampicillin hydrolysis, was evaluated by estimating the inhibition diameter of *S*. *pneumoniae* growth around the hole containing the cell culture.

### β-lactamase activity evaluation in human cells in the presence of β-lactamase inhibitor sulbactam

To evaluate β-lactamase activity in human cells, 10^6^ cell/ml of THP1 cells were incubated on R10 + 2 medium with 0.1 µg/ml of penicillin G alone then infected with the *Pneumococcus* strain and incubated at 37 °C for 24 hours. The same was done with 15 µg/ml sulbactam alone; finally, the THP1 cells were infected with the *Pneumococcus* strain, which was previously incubated in R10 + 2 medium containing 0.1 µg/ml of penicillin G and 15 µg/ml sulbactam. After 24 hours of incubation, the THP1 cells were centrifuged to collect the cell pellet. The latter was washed several times to completely eliminate the antibiotic outside the cells. The collected cells were then lysed. Several dilutions (from 10^−1^ to 10^−6^) of the lysate were cultured on COS agar medium for 24 hours at 37 °C, 5% CO_2_. Following 24 hours of incubation, the bacterial colonies that had grown on the agar plates were counted under the three different conditions tested. The experiment was performed twice in duplicate.

### Spectrophotometry assay for the detection of β-lactamase activity in human cells

THP1 and MRC5 cell pellets were resuspended in 500 µL PBS buffer at a concentration of 10^6^ cell/ml and were flash-frozen in liquid nitrogen. After undergoing three freeze-thaw cycles, the cells were disrupted by three sonication steps (20 seconds, amplitude 50) performed on a Q700 sonicator system equipped with a Cup Horn (QSonica, Newtown, Connecticut, US). Cell debris was discarded following a centrifugation step (15,000 g, 10 minutes). Degradation of the nitrocefin substrate was monitored using a Synergy HT microplate reader (BioTek, USA). Reactions were performed at 25 °C in a 96-well plate in PBS buffer and 5% DMSO with a final volume of 100 µL for each well. The time course hydrolysis of nitrocefin (0.5 mM) was monitored for 30 minutes after adding 50 µL of cell lysate, measuring absorbance at 486 nm.

### Protein expression and purification

Genes encoding for human metallo-ß-lactamase enzymes (i.e. LactB2, MBLAC2, SNM1A, and SNMA1B) were optimized for protein expression in *Escherichia coli* and synthesized by GenScript (Piscataway, NJ, USA). SNM1A and SNM1B could not be obtained in soluble fractions, therefore genes of core MBL-β-CASP domains of SNM1A (aa 676–1040) and SNM1B (aa 1–335) were synthesized, as those were previously described as soluble^28^. The optimized genes were cloned into the pET24a(+) expression plasmid. Recombinant β-lactamases were expressed in *E*. *coli* BL21(DE3)-pGro7/GroEL (TaKaRa) using ZYP-5052 media. Each culture was grown at 37 °C until it reached an OD_600nm_ = 0.8, followed by the addition of L-arabinose (0.2% m/v) and induction, with a temperature transition to 16 °C over 20 hours. Cells were harvested by centrifugation (5000 g, 30 minutes, 4 °C), and the resulting pellets were resuspended in Wash buffer (50 mM Tris pH 8, 300 mM NaCl) and stored at −80 °C overnight. Frozen cells were thawed and incubated on ice for one hour after adding lysozyme, DNase I and PMSF (phenylmethylsulfonyl fluoride) to final concentrations of 0.25 mg/ml, 10 µg/ml and 0.1 mM, respectively. The partially lysed cells were disrupted by three consecutive cycles of sonication (30 seconds, amplitude 45) performed on a Q700 sonicator system (QSonica). The cell debris was discarded following a centrifugation step (10,000 g, 20 minutes, 4 °C). Recombinant β-lactamase proteins were purified using Strep-tag affinity chromatography (wash buffer: 50 mM Tris pH 8, 300 mM NaCl and elution buffer: 50 mM Tris pH 8, 300 mM NaCl, 2.5 mM desthiobiotin) on a 5 ml StrepTrap HP column (GE Healthcare). Fractions containing each protein of interest were pooled. Protein expression and purity were assessed using a 12.5% SDS-PAGE analysis (Coomassie stain). Bands matching protein masses of interest were submitted to mass-spectrometry analysis that confirmed the expression of each desired protein. Protein concentrations were measured using a Nanodrop 2000c spectrophotometer (Thermo Scientific).

### Monitoring β-lactam degradation by Liquid Chromatography-Mass Spectrometry (LC-MS)

Water and acetonitrile solvents were ULC-MS grade (Biosolve). Penicillin G and sulbactam stock solutions at 10 mg/ml were freshly prepared in water from the corresponding high purity salts (Sigma Aldrich). A 1X phosphate-buffered saline (PBS) solution at pH 7.4 was prepared in water from a commercial salt mixture (bioMerieux). Pure solutions of MBLAC2, SNM1A (part of the protein from 676 to 1040 aa) and SNM1B (part of the protein from 1 to 1335 aa) enzymes were buffer-exchanged in PBS, and their concentration was adjusted to 1 mg/ml. THP1 and MRC5 cells from cultures were suspended in PBS at 10^6^ cell/ml. After undergoing three freeze-thaw cycles, the cells were disrupted by three sonication steps (20 seconds, amplitude 50) performed on a Q700 sonicator system equipped with a Cup Horn (QSonica). The cellular debris was discarded following a centrifugation step (15,000 g, 10 min). For each tested enzyme or cell lysate solution, 30 μL was then spiked with penicillin G and sulbactam at a final concentration of 10 μg/ml. Negative controls consisted of PBS spiked with penicillin G and sulbactam Several solutions were prepared to measure metabolites at different incubation times at room temperature. Each time point corresponded to triplicate sample preparations. Then, 70 μL of acetonitrile was added to each sample, and tubes were vortexed 10 minutes at 16000 g to precipitate proteins. The clear supernatant was collected for analysis using an Acquity I-Class UPLC chromatography system connected to a Vion IMS Qtof ion mobility-quadrupole-time of flight mass spectrometer. For each sample, 5 μL stored at 4 °C was injected into a reverse phase column (Acquity BEH C18 1.7 μm 2.1 × 50 mm, Waters) maintained at 50 °C. Compounds were eluted at 0.5 ml/min using water and acetonitrile solvents containing 0.1% formic acid. The following composition gradient was used: 10–70% acetonitrile within 3 minutes, 95% acetonitrile for a 1-minute wash step, and back to the initial composition for 1-minute. Compounds were ionized in the positive mode using a Zspray electrospray ion source with the following parameters: capillary/cone voltages 3 kV/80 V, and source/desolvation temperatures 120/450 °C. Ions were then monitored using a High Definition MS(E) data independent acquisition method with the following settings: travelling wave ion mobility survey, 50–1000 m/z, 0.1 s scan time, 6 eV low energy ion transfer, and 20–40 eV high energy for collision-induced dissociation of all ions (low/high energy alternate scans). Mass calibration was adjusted within each run using a lockmass correction (Leucin Enkephalin 556.2766 m/z). The Vion instrument ion mobility cell and time-of-flight tube were calibrated beforehand using a Major Mix solution (Waters) to calculate collision cross section (CCS) values from ion mobility drift times and mass-to-charge ratios. 4D peaks, corresponding to a chromatographic retention time, ion mobility drift time and parents/fragments masses, were then collected from raw data using UNIFI software (version 1.9.3, Waters). As reported, penicillin G can be degraded in alkaline or acidic pH and in the presence of β-lactamase into different metabolites, including benzyl penilloic acid or benzylpenillic acid. A list of known chemical structures, including penicillin G and its metabolites^18,29^, were targeted with the following parameters: 0.1 minutes retention time window, 5% CCS tolerance, 5 ppm m/z tolerance on parent adducts (H+ and Na+) and 10 mDa m/z tolerance on predicted fragments. Retention times and CCS values were previously measured from penicillin G degradation experiments at pH 2 and pH 10 in order to perform subsequent accurate structures screening. Detector counts of the targeted structures were then collected for data interpretation.

### β-Lactamase activity detection and inhibition assays on purified enzymes

Purified recombinant β-lactamases previously obtained were submitted to a BBL™ Cefinase™ paper disc test^30^ (Becton Dickinson). All protein samples were adjusted to a final concentration of 2 mg/ml. For each recombinant β-lactamase, 15 µL was deposited onto a paper disc impregnated with nitrocefin and incubated at room temperature. The negative control was 15 µL of extracted proteins from induced BL21(DE3)-pGro7/GroEL strains that did not contain any β-lactamases genes. When a change of colour from yellow to red was noticeable within 30 minutes of incubation, corresponding to hydrolysis of the amide bond in the β-lactam ring of nitrocefin, we considered the tested fraction to contain an active β-lactamase enzyme. Degradation of the nitrocefin substrate was also monitored using a Synergy HT microplate reader (BioTek, USA). Reactions were performed at 25 °C in a 96-well plate in PBS buffer and 5% DMSO with a final volume of 100 µL for each well. Time course hydrolysis of nitrocefin (0.5 mM) was monitored for 10 minutes after adding 50 µL of previously prepared protein sample, with absorbance at 486 nm. For the inhibition assay, active β-lactamases at a final concentration of 0.5 mg/ml were briefly incubated with 0.1 mM sulbactam. Negative controls with only sulbactam in buffer and positive controls containing enzymes without any inhibitor were also prepared. After adding 0.5 mM nitrocefin, its hydrolysis was monitored over time with absorbance at 486 nm.

Moreover, the hydrolysis activity of expressed hMBLs was tested on penicillin G microbiologically using a *Pneumococcus* strain highly susceptible to penicillin G (MIC = 0.012 µg/ml) and resistant to sulbactam (MIC = 24 µl/ml). For this purpose, different solutions including penicillin G alone, sulbactam alone, MBLAC2 alone, MBLAC2 + penicillin G, MBLAC2 + penicillin G + sulbactam, were prepared and incubated at room temperature for two hours. 30 µl of each of these solutions was then added into different holes made on an agar plate previously mapped with the suspension of *Pneumococcus* strain at a density of 0.5 MacFarland. Degradation of penicillin G was then evaluated by the ability of the highly penicillin G-susceptible *Pneumococcus* to grow on the agar plate, around each hole.

### Purified β-lactamase enzymes kinetic characterization

Kinetic assays were monitored using a Synergy HT microplate reader (BioTek, USA). Reactions were performed at 25 °C in a 96-well plate (6.2 mm path length cell) in buffer, 50 mM Tris pH 8, 300 mM NaCl, and 5% DMSO, with a final volume of 100 µL for each well. The time course hydrolysis of nitrocefin (ε_486nm_ = 20500 M−1.cm−1) with final concentrations varying between 0.05 and 1.5 mM was monitored for 10 minutes with absorbance at 486 nm, corresponding to the appearance of a red product. Enzymes were kept at a final concentration of 0.3 mg/ml for kinetic studies. For each substrate concentration, the initial velocity was evaluated by Gen5.1 software. The mean values obtained were fitted using the Michaelis-Menten equation on GraphPad Prism 5 software to determine the catalytic parameters.

## Supplementary information

Supplementary informations
