# Supplementary material for: Human metallo-β-lactamase enzymes degrade penicillin
Source: Sci Rep. 2019 Aug 21;9:12173. doi: 10.1038/s41598-019-48723-y (PMC6704141; doi:10.1038/s41598-019-48723-y)
Supplement: Supplementary file 1 — Supplementary informations [file 41598_2019_48723_MOESM1_ESM.doc]

**Human metallo-β-lactamase enzymes degrade penicillin**

Seydina M. Diene^1**^, Lucile Pinault^2**^, Vivek Keshri^1-3^, Nicholas Armstrong^2^, Saber Khelaifia^3^, Eric Chabrière^1^, Gustavo Caetano-Anolles^4^, Philippe Colson^1-2^, Bernard La Scola^1-2^, Jean-Marc Rolain^1-2^, Pierre Pontarotti^1-5^, Didier Raoult^1-2*^

Corresponding author: Prof. Didier Raoult, Email: [didier.raoult@gmail.com](mailto:didier.raoult@gmail.com)


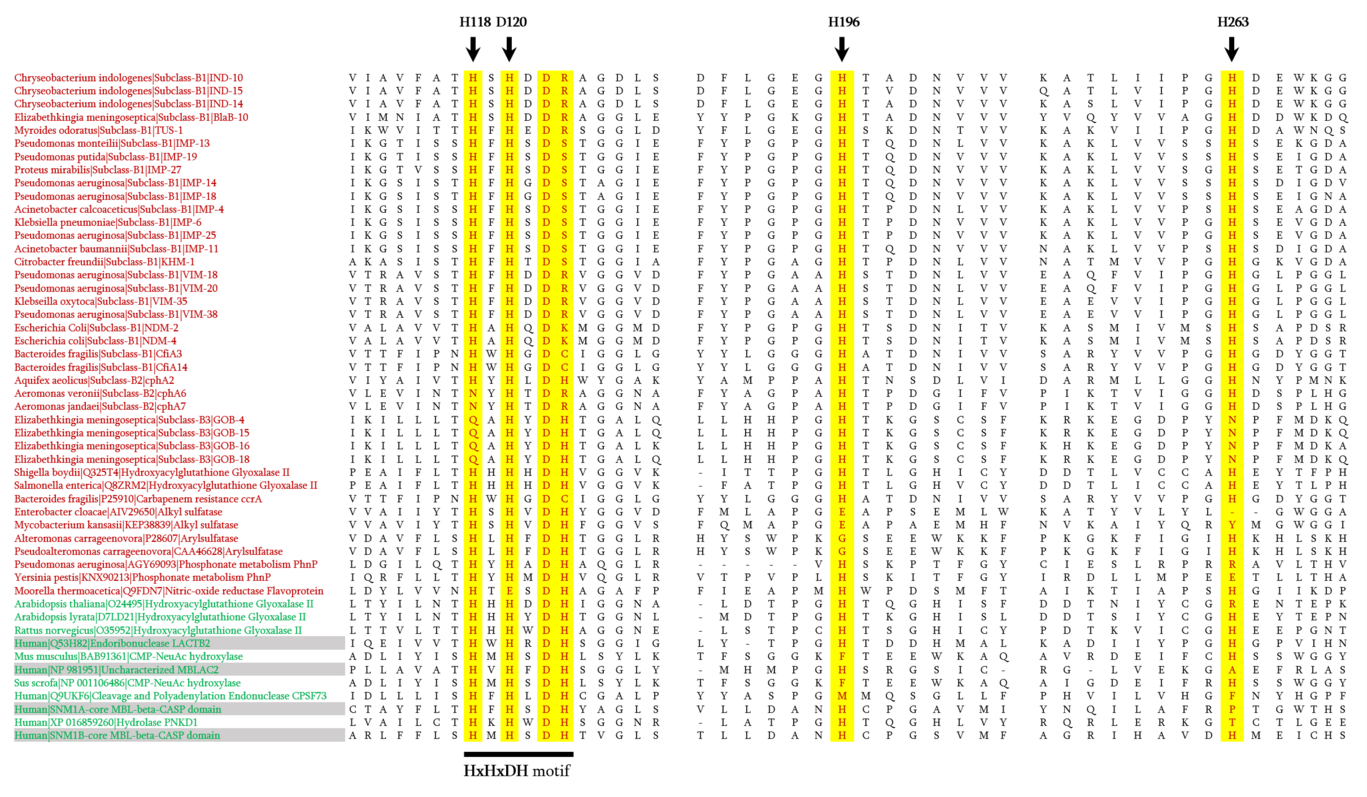


**Supplementary Fig. 1: Alignment of MBL protein sequences from bacteria (red) and eukaryotes (green)**. The conserved residues/motifs are highlighted in yellow. The highlighted sequences (in grey) refer to the synthetized and experimentally tested enzymes in this study.


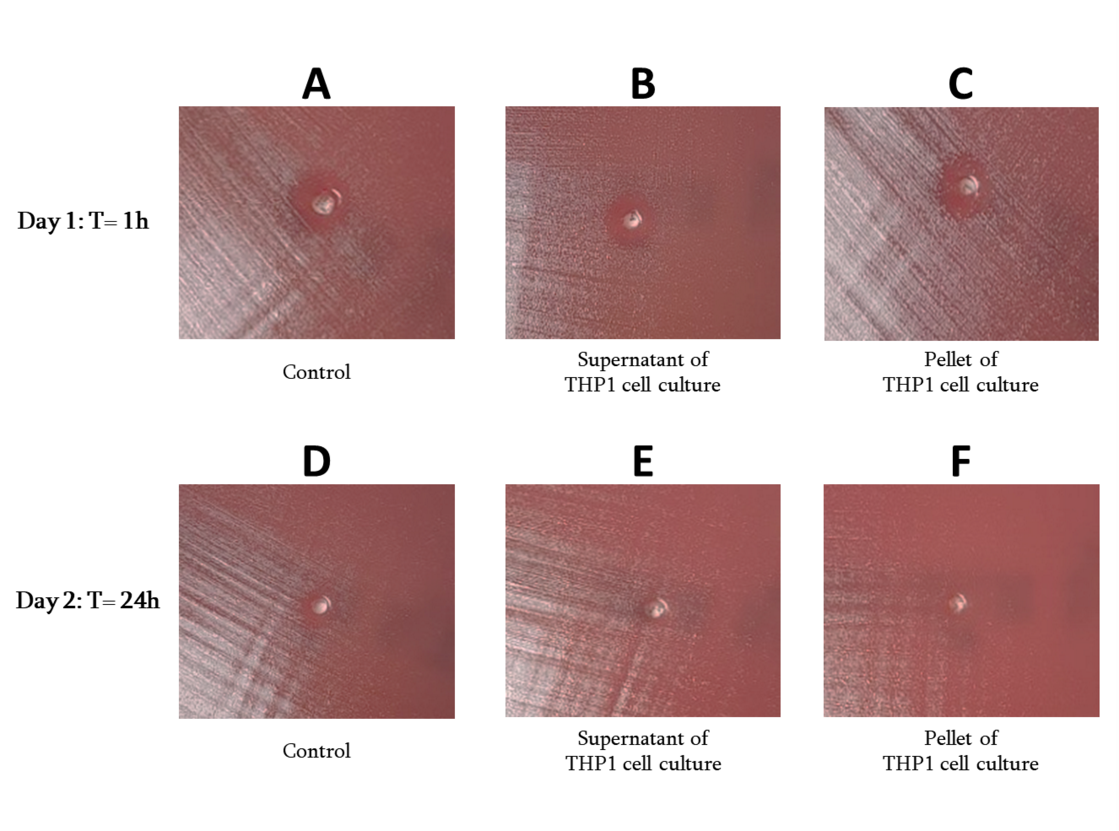


**Supplementary Fig. 2:** **Evaluation of ampicillin degradation in a human THP1 cell culture.** Antibiotic degradation is evaluated here by the growth of a highly ampicillin susceptible *S. pneumoniae* strain around the hole containing a pellet or supernatant of human cells incubated with 2 µg/ml of ampicillin. Antibiotic degradation was confirmed when the *S. pneumoniae* strain was able to grow in contact with the hole, as shown in pictures **E** and **F** after 24 hours.


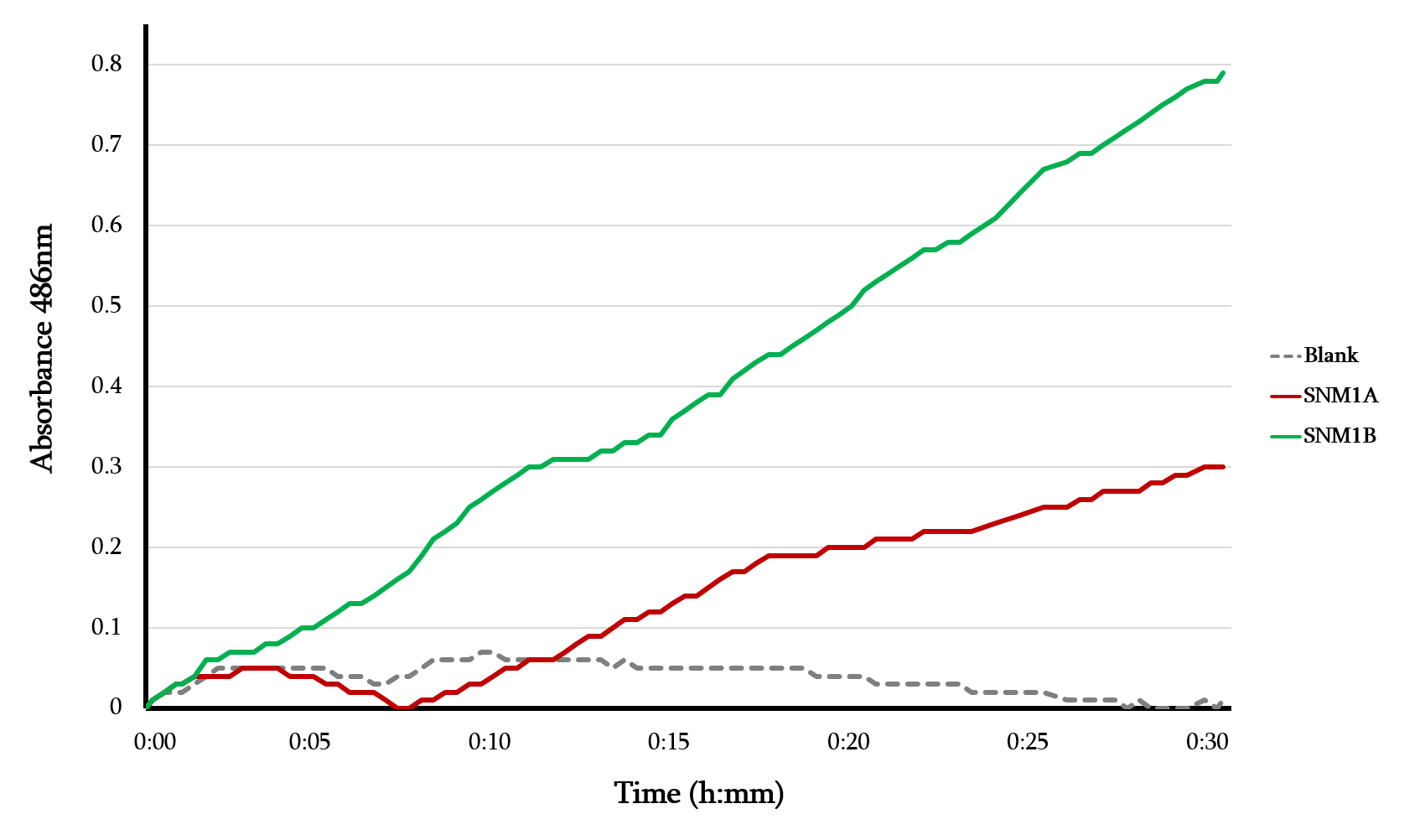


**Supplementary Fig. 3:** Monitoring the activity of SNM1A and SNM1B hMBL enzymes by spectrophotometric assay of nitrocefin degradation in the presence of both enzymes during 30 minutes.


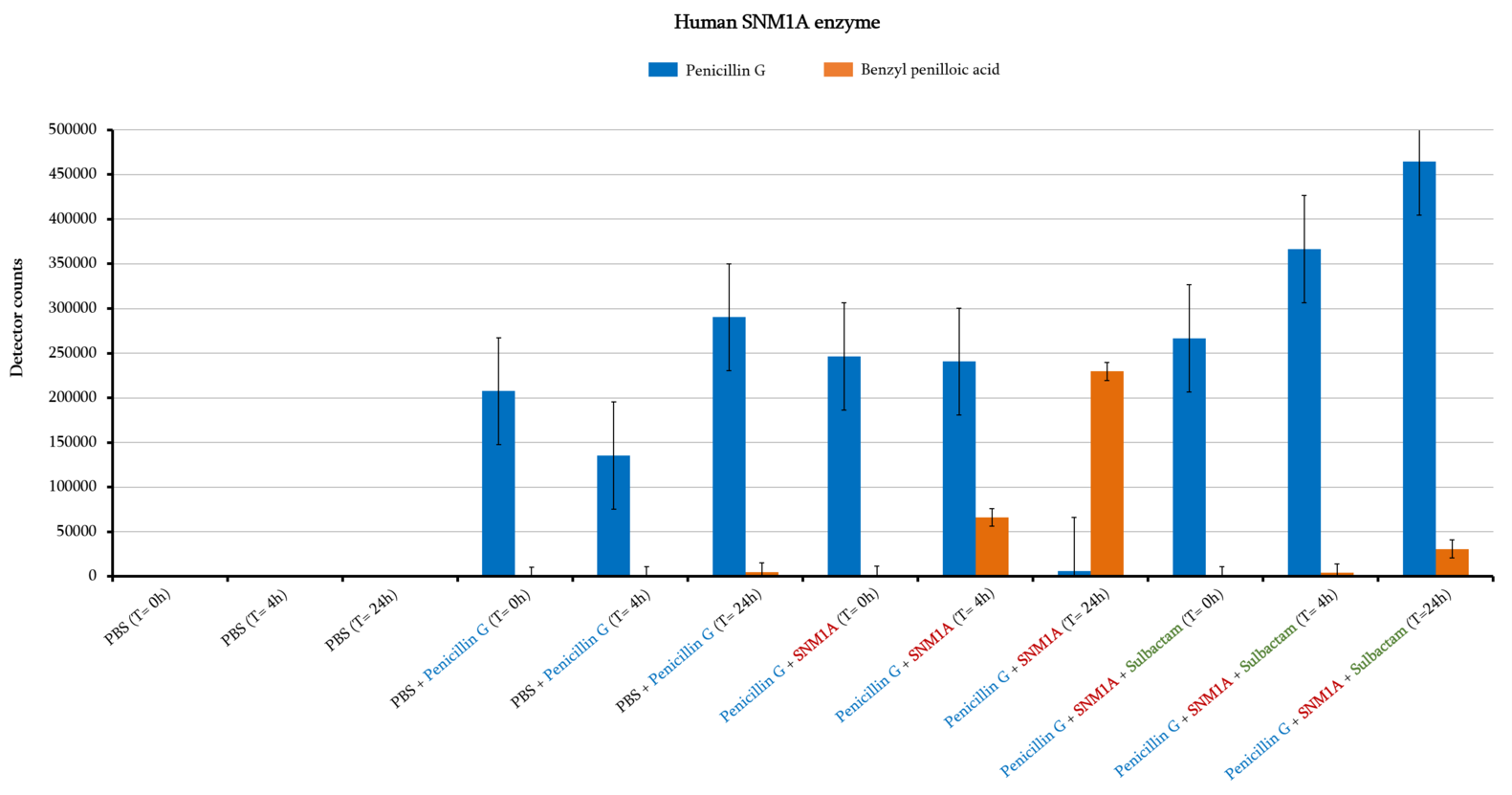


**Supplementary Fig. 4:** Determining the human SNM1A activity on penicillin G in presence and absence of sulbactam by LC/MS average detector counts of metabolite benzyl penilloic acid (the penicillin G metabolite) monitored during 24 hours.


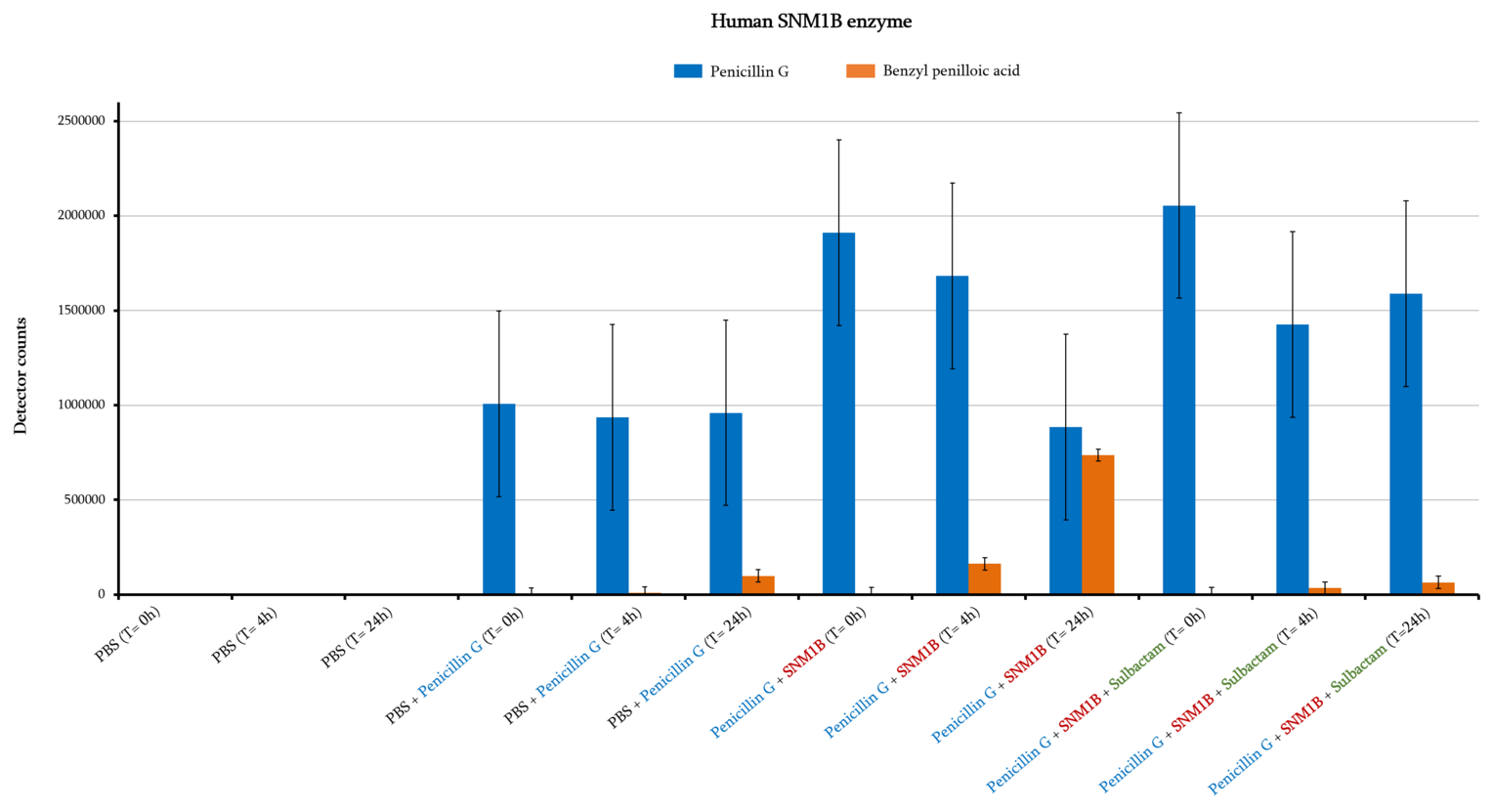


**Supplementary Fig. 5:** Determining the human SNM1B activity on penicillin G in presence and absence of sulbactam by LC/MS average detector counts of metabolite benzyl penilloic acid (the penicillin G metabolite) monitored during 24 hours.

**Supplemental** **Table 1:** Three-dimensional (3D) structure comparison of the tested hMBLs with available and characterized proteins from the Phyre^2^ investigator database.

| **Protein sequences** | **Size (aa)** | **Phyre^2^ investigator database** | | | | | |
| --- | --- | --- | --- | --- | --- | --- | --- |
|  |  | **Best protein hit** | **Hit ID** | **Confidence** | **Coverage** | **Comments** | **3D Structure** |
| hMBL  **LactB2** endonuclease | 288 | Crystal structure of human β-lactamase-like protein 2 (LactB2) | C4ad9E | 100 % | 100 % | 288 residues (100% of sequence) have been modelled with 100% confidence by the single highest scoring template. | 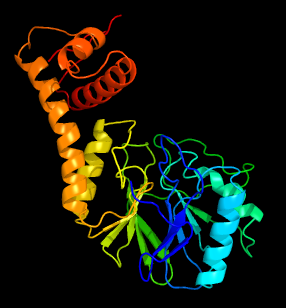 |
| hMBL  **MBLAC2** metallo-β-lactamase | 258 | Crystal structure of a beta-lactamase domain protein from *Burkholderia ambifaria* | C5i0pB | 100 % | 92 % | 258 residues (92% of sequence) have been modelled with 100.0% confidence by the single highest scoring template | 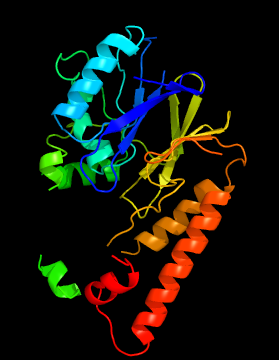 |
| hMBL  **SNM1A**-core MBL-beta-CASP domain | 365 | Crystal structure of human dna cross-link repair 1a (Hydrolase) | C4b87A | 100% | 92% | 335 residues (92% of your sequence) have been modelled with 100.0% confidence by the single highest scoring template | 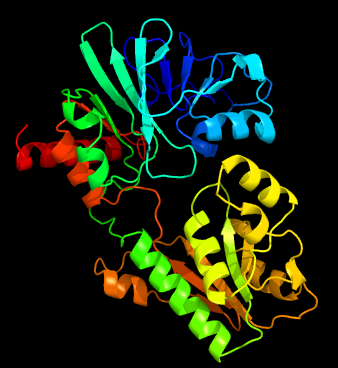 |
| hMBL  **SNM1B**-core MBL-beta-CASP domain | 335 | Crystal structure of human 5' exonuclease Apollo (Hydrolase) | C3zdkA | 100% | 93% | 313 residues (93% of your sequence) have been modelled with 100.0% confidence by the single highest scoring template | 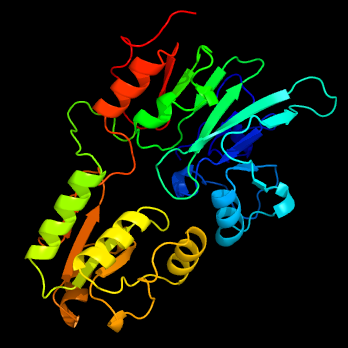 |

**Supplemental** **Table 2**: Kinetic values of the enzymatic activity of the described β-lactamases compared to that of the human MBLAC2 β-lactamase.

|  | **Nitrocefin*** | | | | |
| --- | --- | --- | --- | --- | --- |
|  | kcat (s-1) |  | Km (µM) |  | kcat/Km (M-1 S-1) |
| **MBL Subclass B1** |  |  |  |  |  |
| BcII | 45 |  | 70 |  | 6.4E+05 |
| CcrA | 200 |  | 20 |  | 1E+07 |
| BlaB | 20 |  | 70 |  | 2.8E+05 |
| **Acquired-B1** |  |  |  |  |  |
| IMP-1 | 30 |  | 30 |  | 2E+06 |
| VIM-1 | 100 |  | 20 |  | 5E+06 |
| SPM-1 | 0.05 |  | 4 |  | 1.2E+05 |
| GIM-1 | 6 |  | 10 |  | 6E+05 |
| **MBL Subclass B2** |  |  |  |  |  |
| CphA | 0.003 |  | 1200 |  | 2.5 |
| ImiS | 0.06 |  | 20 |  | 3E+03 |
| **MBL Subclass B3** |  |  |  |  |  |
| L1 | 20 |  | 10 |  | 2E+06 |
| FEZ-1 | 90 |  | 100 |  | 9E+05 |
| GOB-1 | - |  | - |  | - |
| Mbl1b | 1800 |  | 100 |  | 1.8E+07 |
| **Human Metallo-Beta-lactamase MBLAC2** | **0.0004** |  | **370.5** |  | **1.12** |

* These values are obtained from Bebrone *et al Biochem. Pharmacol.* **74**, 1686–1701 (2007)*.*
